# Toscana virus isolated from sandflies, Morocco

**DOI:** 10.1186/s13071-015-0826-1

**Published:** 2015-04-03

**Authors:** Nargys Es-sette, Malika Ajaoud, Latifa Anga, Fouad Mellouki, Meryem Lemrani

**Affiliations:** Laboratoire de Parasitologie et Maladies Vectorielles, Institut Pasteur du Maroc, Casablanca, Morocco; Laboratoire de Chimie Physique et Bioorganique, URAC C22, Faculté des Sciences et Techniques, Université Hassan II Casablanca, Mohammedia, Morocco; Laboratoire de Virologie Médicale, Institut Pasteur du Maroc, Casablanca, Morocco

**Keywords:** Toscana virus, Sandflies, *Phlebotomus longicuspis*, *Phlebotomus sergenti*, Morocco

## Abstract

To investigate the transmission of phleboviruses, a total of 7,057 sandflies were collected in well-known foci of cutaneous leishmaniasis and were identified to species level according to morphological characters.

Collected sandflies were tested by Nested PCR for the presence of Phleboviruses and subsequently by viral isolation on Vero cells. The corresponding products were sequenced. Toscana virus was isolated, for the first time, from 5 pools of sandflies.

Hence, Toscana virus should be considered a potential risk that threatens public health and clinicians should be aware of the role of Toscana virus in cases of meningitis and encephalitis in Morocco.

## To the Editor

Toscana virus (TOSV) (family *Bunyaviridae,* genus *Phlebovirus,* species *Sandfly Naples virus*) is an arthropod-borne virus transmitted by sandflies. It was shown to be endemic in many countries surrounding the Mediterranean and is an interesting example of an emergent virus transmitted by sandflies. To date, TOSV differs from other phleboviruses with its neurotropism; it is the only sandfly-borne phlebovirus to be unambiguously associated with central nervous system manifestations. Asymptomatic infection and infection without central nervous system involvement due to TOSV, such as febrile erythema or influenza-like illness, has also been described [1].

TOSV is transmitted to humans by *Phlebotomus*, *Sergentomyia* genera, frequently by *Phlebotomus perniciosus*, *P. perfiliewi*, and *S. minuta* [2-4].

TOSV has also been isolated from the brain of a bat in areas where *P. perniciosus* and *P. perfiliewi* were present [4]*.* In Morocco, TOSV has recently been detected in sandflies [5,6] but never isolated.

A total of 7,057 sandflies were collected in the period between 2008 and 2011 by using CDC light traps in two well-known foci of cutaneous leishmaniasis (CL) (province of Azilal and province of Sefrou) situated in different bioclimatic areas.

Sandflies were identified by their morphological characteristics. Each sand fly was dissected under a binocular microscope, on a sterilized microscopic slide using sterile steel entomological needles. The head and genitalia of each sand fly were mounted under a cover slip in Marc-André solution for morphological identification at the species level according to morphological keys described by the Moroccan Health Ministry [7]. After dissection, the abdomen and the thorax of each female specimen were transferred to sterile 1.5 ml Eppendorf tube and then stored at −80°C pending examination.

In Sefrou province, the sandflies collected consisted of 10 species, of which seven belonged to the genus *Phlebotomus* and three to the genus *Sergentomyia*. The most abundant species was *P. longicuspis*, accounting for 72% of the total sandflies collected (Table 1). In Azilal province, 10 species belonging to the genus *Phlebotomus* and 3 belonging to the genus *Sergentomyia* were identified. *P. sergenti* is the most abundant, in the whole focus its relative abundance is 47.32% (Table 1).

**Table 1:** **Number of specimens of males and females identified, the abundance and the number of pools tested for each species**

**Species**	**Azilal**						**Sefrou**					
	**F**	**%**	**Number of pools**	**M**	**%**	**Number of pools**	**F**	**%**	**Number of pools**	**M**	**%**	**Number of pools**
***P. sergenti***	965	48	32	1051	44	33	286	19	11	271	24	11
***P. perniciosus***	374	19	11	808	34	26	50	3	2	85	8	3
***P. longicuspis***	421	21	16	206	9	7	1147	75	44	743	66	30
***P. papatasi***	141	7	5	146	6	5	30	2	2	6	1	1
***P. perfiliewi***	5	0	1	0	0	0		0	0	0	0	0
***P. ariasi***	3	0	1	0	0	0	4	0	1	1	0	1
***P. chabaudi***	3	0	1	0	0	0	1	0	1	0	0	1
***P. kazeruni***	0	0	0	1	0	1	0	0	0	0	0	0
***P. langeroni***	0	0	0	2	0	1	10	1	1	10	1	1
***P. bergeroti***	0	0	0	0	0	0	0	0	0	0	0	0
***P. chadlii***	0	0	0	1	0	1	0	0	0	0	0	0
***P. alexandri***	0	0	0	0	0	0	0	0	0	0	0	0
***S. minuta***	63	3	3	16	1	1	3	0	1	0	0	0
***S. antennata***	5	0	1	17	1	1	0	0	0	2	0	1
***S. fallax***	27	1	2	19	1	1	0	0	0	0	0	0
***S. dreyfussi***	0	0	0	0	0	0	1	0	1	0	0	0
**Total**	2007	100	73	2400	100	77	1532	100	64	1118	100	49

The monospecific pools were homogenized and supernatants were used for virus isolation in Vero cells. Viral RNA was extracted by using the “Mini kit MACHEREY-NAGEL Nucleospin RNA II”, according to the manufacturer’s protocol. Nested polymerase chain reactions (PCR) were performed by using degenerated primers specific for regions of the polymerase (large [L]) genes [8].To avoid the risks of contamination, positive controls were not included in the experiment.

Five out of 235 pools showed a cytopathic effect in Vero cell cultures and phlebovirus RNA was detected by nested PCR in the 5 pools: 2 pools of *P. longicuspis* (1 female and one male) in Sefrou province, while in Azilal province 3 pools of *P. sergenti* male were positives.

Positive pools were sequenced on both strands by using forward and reverse primers for the L gene [8].

TOSV was obtained from the 5 pools of sandflies. Resulting sequences were aligned together with homologous sequences of selected members of the genus Phlebovirus retrieved from GenBank database. Analysis was performed by using CLUSTAL in DAMBE software, and sequences were analyzed in MEGA version 6. Complete genome sequencing is ongoing by using the Ion PGM Sequencer in UMR190 “Emergence des Pathologies Virales in Marseille.

**Figure 1:**
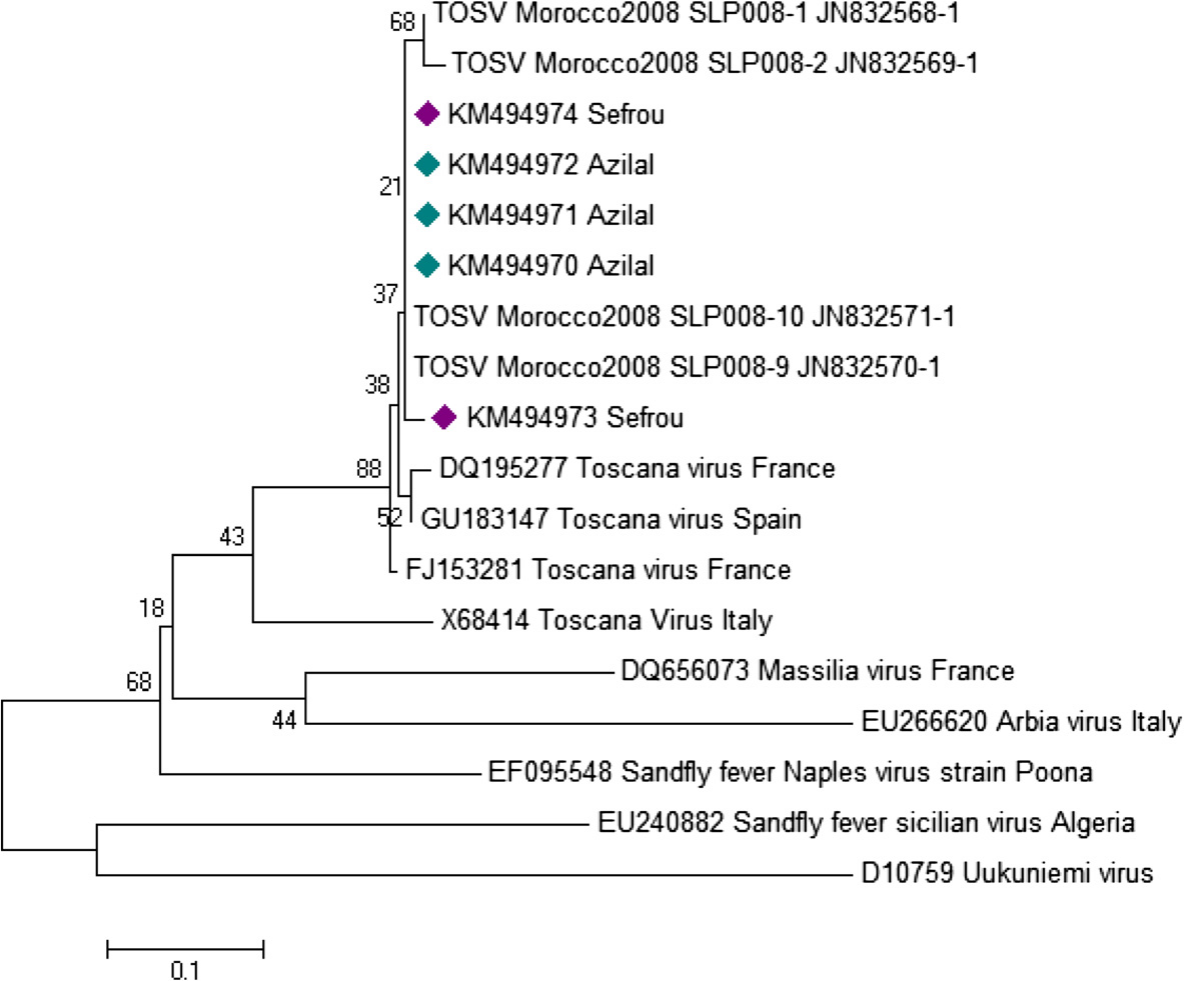
**Phylogenetic analysis of TOSV of Morocco based on nucleotide in the L-polymerase gene.** The evolutionary history was inferred using the Neighbor-Joining method. The optimal tree with the sum of branch length = 1.82678464 is shown. The percentage of replicate trees in which the associated taxa clustered together in the bootstrap test (500 replicates) are shown next to the branches. The tree is drawn to scale, with branch lengths in the same units as those of the evolutionary distances used to infer the phylogenetic tree. The evolutionary distances were computed using the Maximum Composite Likelihood method and are in the units of the number of base substitutions per site. The analysis involved 17 nucleotide sequences. Evolutionary analyses were conducted in MEGA6. Bullet points indicate virus names that correspond to sequences determined in this study (Green bullet points in Azilal province and blue bullet points in Sefrou province).

Phylogenetic analyses of the L gene indicated that those viruses clustered with TOSV strains circulating in Morocco, Spain and France (Figure 1). TOSV detected in this study belongs to the genotype B, previously recognized in France and Spain, countries geographically close to Morocco.

Thus, the presence of *P. sergenti*, vector of *L. tropica* in Azilal province [9] and *P. longicuspis*, vector of *L. infantum* in Sefrou [10] province makes those foci of high risk, as the two phlebotomine species are incriminated in the transmission of TOSV as well as *L. tropica* and *L. infantum* causative agents of cutaneous and visceral leishmaniasis respectively.

*P. longicuspis* has never been involved in the transmission of TOSV. However, further studies are needed to confirm the transmission of this phlebovirus by *P. longicuspis*.

In conclusion, these foci of CL, where Phleboviruses and *Leishmania* co-exist, should be considered at potential risk, because persons exposed to *Leishmania* parasite infection are at greater risk of being infected with TOSV and *vice versa*.

This finding calls for further studies of full-genome sequencing as well as serologic characterization to monitor distribution of vector-borne diseases, by analyzing patient samples with summer fever or unknown infections of the central nervous system.

## Ethical approval

The study protocol was approved by the Committee on Research Ethics of the Institut Pasteur du Maroc.
